# Eye position effects on the remapped memory trace of visual motion in cortical area MST

**DOI:** 10.1038/srep22013

**Published:** 2016-02-23

**Authors:** Naoko Inaba, Kenji Kawano

**Affiliations:** 1 Department of Integrative Brain Science, Graduate School of Medicine, Kyoto University. Kyoto 606-8501, Japan; 2 Department of Physiology, Hokkaido University School of Medicine. Sapporo 060-8638, Japan

## Abstract

After a saccade, most MST neurons respond to moving visual stimuli that had existed in their post-saccadic receptive fields and turned off before the saccade (“trans-saccadic memory remapping”). Neuronal responses in higher visual processing areas are known to be modulated in relation to gaze angle to represent image location in spatiotopic coordinates. In the present study, we investigated the eye position effects after saccades and found that the gaze angle modulated the visual sensitivity of MST neurons after saccades both to the actually existing visual stimuli and to the visual memory traces remapped by the saccades. We suggest that two mechanisms, trans-saccadic memory remapping and gaze modulation, work cooperatively in individual MST neurons to represent a continuous visual world.

Because of saccadic eye movements, images of the visual scene rapidly sweep across the retina and thereby shift to different retinal locations. Despite such blurring and displacement of the retinal images, we are able to perceive the visual world as stable and continuous. Saccadic suppression is thought to be an important mechanism that compensates for the blurred retinal images^1^. The visual images obtained after each retinal image shift must be updated in the correct coordinates to construct a stable and continuous representation of the visual world. Two neural mechanisms have been proposed that address the saccade displacement problem^2^. One mechanism is known as trans-saccadic remapping, in which neurons shift their receptive fields (RFs), providing anticipatory activity in accordance with each saccade, and referred to as “predictive remapping.” There are several visual areas in which neurons exhibit predictive RF shifts to the location into which the saccade brings the stimulus, including the lateral intraparietal area (LIP)^3^,^4^,^5^,^6^,^7^, frontal eye field (FEF)^8^,^9^,^10^, superior colliculus (SC)^11^,^12^,^13^, and extrastriate visual areas^14^,^15^.

The second mechanism, spatiotopic representation, occurs via neurons whose visual responses are modulated by eye position. To perceive a continuous and stable visual world despite the displacement, visual images may be represented in spatiotopic coordinates rather than retinotopic coordinates. Neurons whose visual responses are modulated by eye position, so called “eye position gain field”, have been observed in areas LIP, medial superior temporal (MST), middle temporal (MT), ventral intraparietal (VIP), and 7a^16^,^17^,^18^,^19^,^20^,^21^. These areas are hypothesized to form inputs to the spatiotopic map of the visual scene^22^,^23^,^24^.

In this context, the neural correlates of the two proposed mechanisms that address the saccade displacement problem, trans-saccadic remapping and spatiotopic representation have been reported in various areas. However, the relationship and/or interaction between the two mechanisms have not yet been studied. It is also unclear whether the two mechanisms work together in individual neurons. To achieve a continuous and stable representation of the external world, it is very likely that these two mechanisms work cooperatively, potentially within individual neurons.

We recently reported that after a saccade, most MST neurons responded to a visual stimulus that had pre-existed inside their post-saccadic RFs and then turned off before the saccade (i.e., “memory remapping”)^25^. The responses of these neurons were neither predictive nor anticipatory because their responses to the stimulus in the post-saccadic RF were observed only after the saccades. However, their remapped memory traces should be able to contribute to dealing with the saccade displacement problem. In the present study, to understand the characteristics of trans-saccadic memory in spatiotopic coordinates, we investigated the dependence of the remapped memory trace of visual motion on eye position in MST. We found that the visual sensitivity of MST neurons after the saccades was modulated by the gaze angle both to the real visual stimuli and to the visual memory traces remapped by the saccades, suggesting their role in constructing a stable and continuous visual world despite saccadic eye movements.

## Results

We recorded single unit activities in the cortical area MST of three hemispheres of two monkeys and studied their detailed response properties in 164 neurons. All of them responded to large field motion in a directionally selective manner; their average firing rates in the preferred directions were greater than or equal to 1.5 times those in the direction opposite to the preferred directions.

**Figure 1:**
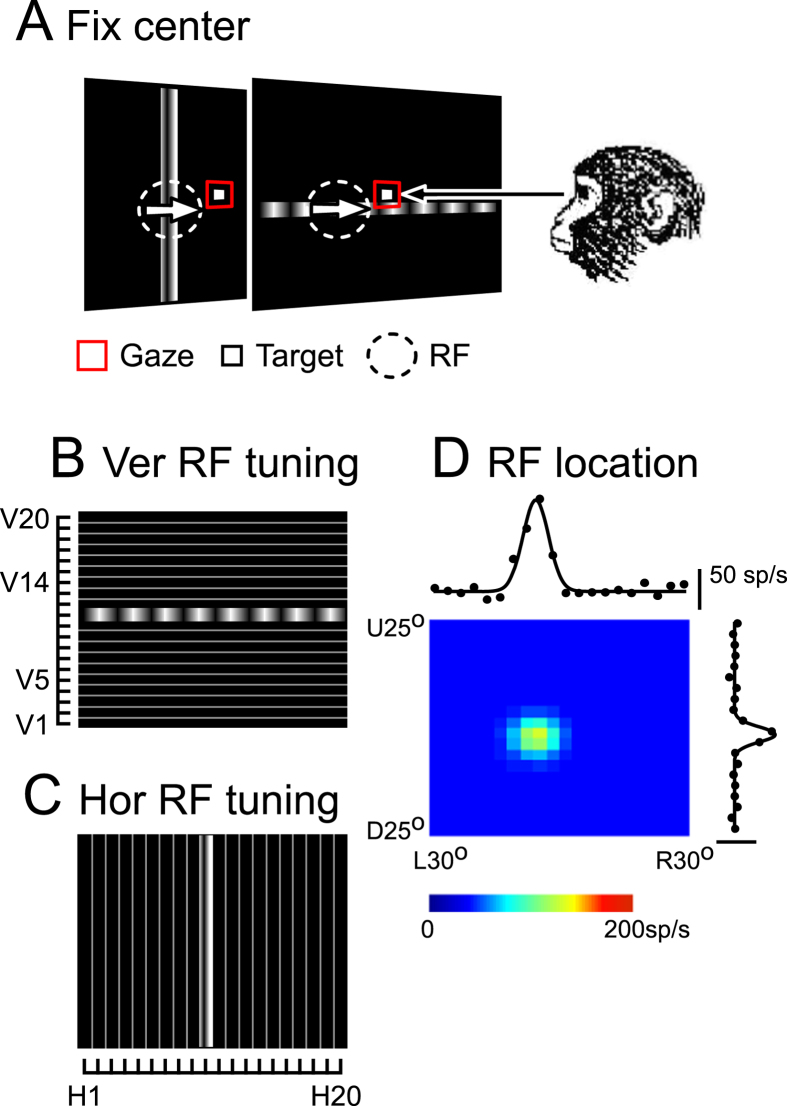
Responses of an MST neuron during central fixation. (**A**) Schematic diagram showing the central fixation task. The monkey was required to keep its eyes fixed on a target at the screen center. A moving grating was presented in a vertical (back panel) or horizontal strip (front panel) and moved in the neuron’s preferred direction (rightward, white arrows). The red square shows the monkey’s gaze location. (**B**,**C**) Schematic diagram showing the screen where the moving grating was presented in a narrow horizontal (2.5° width, (**B**)) or vertical strip (3° width, (**C**)) at one of 20 locations divided vertically ((**B**), V1–V20) or horizontally ((**C**), H1–H20). Note that the orientation of the grating pattern was vertical irrespective of the direction of the strip, because the neuron preferred horizontal (rightward) motion. (**D**) Two-dimensional RF map of an MST neuron during central fixation together with spatial tuning curves (fitted Gaussian functions) of the responses. The color indicates the mean firing rate (sp/s).

To define the RF of each neuron, we observed its responses to a moving grating in a long, narrow horizontal (or vertical) strip, which was presented at one of 20 locations vertically (or horizontally), divided on the CRT screen, during the fixation task (Fig. 1). The mean firing rates at the 20 locations were utilized to calculate the vertical (or horizontal) distribution of the response amplitude as cross-sections of a two-dimensional tuning surface of the RF. A sample two-dimensional RF map of an MST neuron is shown in Fig. 1D. The neuron’s RF covered an area of approximately 17.8° × 15.7°, and its central location deviated left-down (−4.2°, −3.5°) from the center of the screen. It was in the range of the commonly recorded MST neurons’ RF size when the border of the RF can be determined inside the CRT screen of 60° width × 50° height (see Methods and Fig. 5 of Miura, *et al*.^26^).

**Figure 2:**
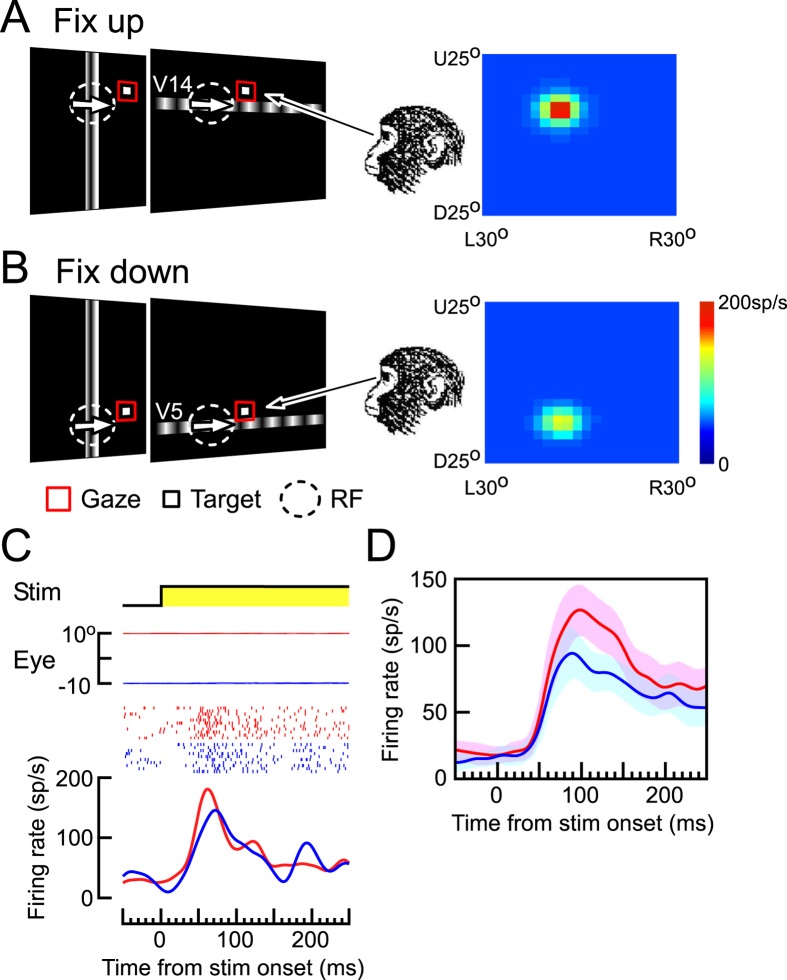
Gaze modulation during fixation. (**A**,**B**) Left: Schematic diagram showing the upward (**A**) and downward (**B**) fixation task. The monkey was required to keep its eyes fixed on a target. The location of the target was at 10° up (**A**) or 10° down (**B**) from the screen center. A moving grating was presented and moved in the neuron’s preferred direction (rightward, white arrows). Right: Two-dimensional RF maps of the same MST neuron during upward (**A**) and downward (**B**) fixation. The color indicates the mean firing rate (sp/s). (**C**) Effects of gaze angle on the responses of the MST neuron. Each panel, from top to bottom, indicates the status of the moving grating (yellow: stimulus-on), vertical eye position (positive: upward), impulse-raster, and spike-density function. The monkey fixated a target located 10° up (red) or 10° down (blue). The location of the moving grating was either at V14 (red, upward fixation) or at V5 (blue, downward fixation). See Fig. 1B for the locations of V5 and V14. (**D**) Population averages of the firing rates of MST neurons (N = 52) after the stimulus onset while the animal kept its gaze on the fixation target located at high- (red) and low-gain field (blue). Shaded regions around the population responses denote ± 2 standard error of the mean (s.e.m.).

Consistent with previous studies^16^,^17^,^19^,^27^, we observed that the visual sensitivity of MST neurons was modulated by the gaze angle. For the neuron shown in Fig. 1, the responses to the moving grating were larger when the monkey fixated a target located 10° up (Fig. 2A, red line and dots in Fig. 2C) than on a target 10° down from the center (Fig. 2B, blue line and dots in Fig. 2C). The retinotopic location of its RF was approximately the same regardless of the eye positions (two-sided Wilcoxon signed-rank, p > 0.05): the centers of the retinotopic location of the RF were (−4.5°, −3.8°) and (−4.5°, −3.5°) when the monkey fixated on the up- and down-targets, respectively (Fig. 2A,B). However, the amplitude of the visual response was modulated by gaze direction. In other words, for this neuron the target location at 10° up was in the high-gain field and that at 10° down was in the low-gain field. We observed the responses of 52 MST neurons while the monkey fixated on the target located at two spatially separate positions (either horizontally or vertically). The visual responses in 69.2% of the neurons were significantly modulated by gaze direction (two-sided Wilcoxon signed-rank, p < 0.05). The comparison between the population averages of the firing rates during fixation on the target located in high- and low-gain field revealed that the gaze modulation almost coincided with the onset of the visual responses (52.6 ± 24.9 ms, Fig. 2D).

**Figure 3:**
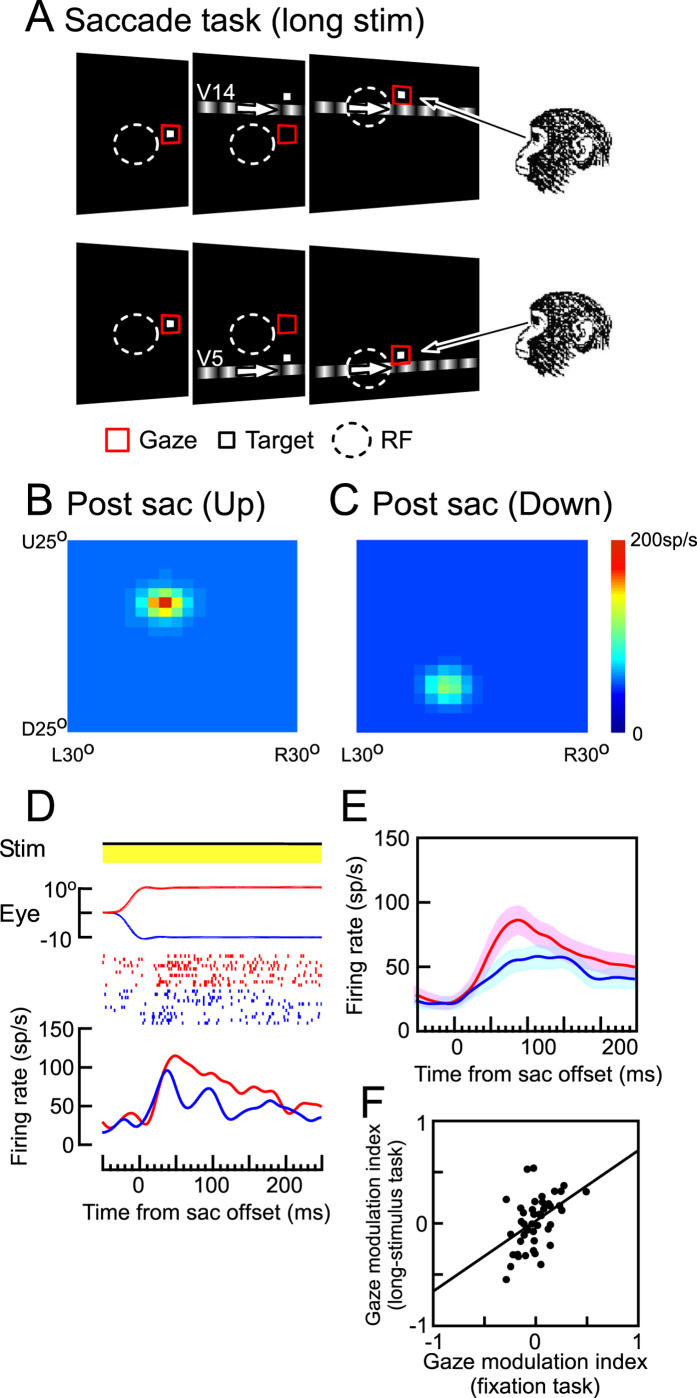
Gaze modulation of post-saccadic responses (long-stimulus task). (**A**) Schematic diagram showing the time sequence of the long-stimulus saccade task. When the monkey maintained central fixation, the fixation target disappeared and a saccade target and moving grating appeared at the same time. The saccade target was located at 10° eccentricity on the vertical (or horizontal) axis (10° up or 10° down in this example). To reduce the effects of the retinal motion induced by saccade itself, the required saccades were parallel to the axis of the grating pattern (vertical in this example). (**B**,**C**) Location of the RF of the same MST neuron after upward (**B**) and downward (**C**) saccades, respectively. The color indicates the mean firing rate (sp/s). (**D**) Effects of gaze angle on the responses of the MST neuron. Each panel, from top to bottom, indicates the status of the moving grating (yellow: stimulus-on), vertical eye position (positive: upward), impulse-raster, and spike-density function. The moving grating was presented for 600 ms and visible before, during, and after saccades. The location of the saccade target was at 10° up and moving grating at V14 (red dots and line, in high-gain field). The location of the saccade target was at 10° down and moving grating at V5 (blue dots and line, in low-gain field). (**E**) Population averages of the firing rates of MST neurons (N = 120) after saccades to the target located at high- (red) and low-gain field (blue). Shaded regions around the population responses denote ± 2 s.e.m. (**F**) Correlation of the gaze modulation indices (GMIs) between the fixation and long-stimulus saccade tasks (N = 51).

Similar response modulations were observed after 10° upward and 10° downward saccades (Fig. 3). In these cases, the moving grating was displayed for 600 ms, and was visible throughout the periods before, during, and after the saccade (Fig. 3A). The neuron increased its firing rate when the eyes arrived at the saccade target and the stimulus fell into its post-saccadic RF (Fig. 3D). The location of the post-saccadic RF on the CRT-screen shifted with eye position changes (Fig. 3B, C). On the retina, the locations of the post-saccadic RF after the upward and downward saccades were very similar: (−4.3°, −2.5°) and (−4.5°, −2.5°), respectively. However, the amplitudes of the post-saccadic responses significantly differed. The responses after the downward saccades were markedly weaker than those after the upward saccades (64.5 ± 28.8 sp/s and 84.5 ± 56.8 sp/s; Fig. 3D). We also observed similar gaze modulation on visual responses during the post-saccadic period in other MST neurons. Significant eye position gain modulation was observed in the post-saccadic responses of 73.2% of the neurons (N = 120 of 164, two-sided Wilcoxon signed-rank, p < 0.05). Again, the comparison between the population averages of the firing rates (N = 120) after the saccades to the target located in high- and low-gain field revealed that the gaze modulation almost coincided with the onset of the responses after the saccades (52.0 ± 24.7 ms, Fig. 3E). The comparison between the gain modulation indices (GMIs) of 51 MST neurons during fixation and after saccade revealed a strong correlation between them, indicating a common gaze modulation mechanism (Pearson’s linear correlation coefficient = 0.43, p < 0.01, Spearman’s rho = 0.42, p < 0.01, Fig. 3F).

**Figure 4:**
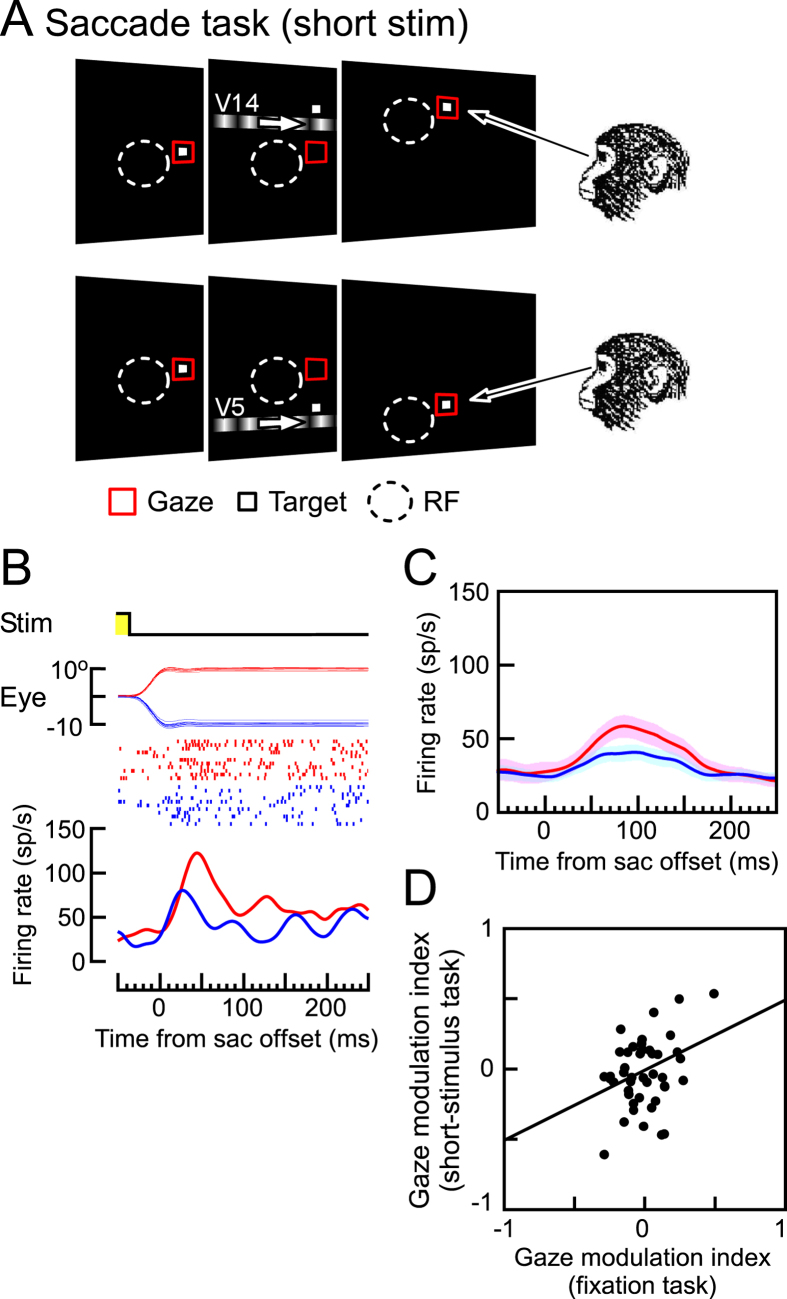
Gaze modulation of the memory response (short-stimulus task). (**A**) Schematic diagram showing the time sequence of the saccade task (short-stimulus task). The moving grating was visible for 170 ms and disappeared before the saccade onset. (**B**) Effects of gaze angle on the responses of the MST neuron. Each panel, from top to bottom, indicates the status of the moving grating (yellow: stimulus-on), vertical eye position (positive: upward), impulse-raster, and spike-density function. The moving grating was presented for 170 ms and turned off before saccades. The location of the saccade target was at 10° up and moving grating at V14 (red dots and line, in high-gain field). The location of the saccade target was at 10° down and moving grating at V5 (blue dots and line, in low-gain field). (**C**) Population averages of the firing rates of MST neurons (N = 119) after saccades to the target located at high- (red) and low-gain field (blue). Shaded regions around the population responses denote ± 2 s.e.m. (**D**) Correlation of the GMIs between the fixation and short-stimulus saccade tasks (N = 51).

In our previous study, we reported that most MST neurons increased their firing rates when a saccade brought the location of the visual stimulus, which had been visible only before the saccade, into their RFs^25^. This finding suggested that the responses of such MST neurons after saccades were evoked by a memory of a pre-existing-but-turned-off stimulus in their post-saccadic RFs (i.e., “memory remapping”). In this situation, the visual stimulus was presented when the eyes were in the pre-saccadic position, which was always on the center of the screen. In contrast, the memory was recalled and the neuronal responses began when the eyes arrived at the post-saccadic position. After the findings of our previous study, we aimed to examine the effect of gaze angle on the remapped memory traces using short-duration visual stimuli, which were visible for 170 ms and disappeared before the saccade (Fig. 4A). We found that the responses after the downward saccades (46.4 ± 32.3 sp/s; blue line in Fig. 4B) were significantly weaker than those after the upward saccades (78.2 ± 44.0 sp/s; red line in Fig. 4B). This indicates that the modulation of the memory responses reflected the post-saccadic eye position. In most of the MST neurons (119 of 143), modulation of the remapped memory responses by the eye position was also observed after the saccade (two-sided Wilcoxon signed-rank, p < 0.05). The comparison between the population averages of the firing rates (N = 119) after the saccades to the target located in high- and low-gain field revealed that the gaze modulation of the remapped responses almost coincided with the onset of the remapped responses (50.6 ± 25.0 ms, Fig. 4C). A significant correlation between the GMIs of 51 MST neurons during fixation and remapped responses also indicates a common gaze modulation mechanism between them (Pearson’s linear correlation coefficient = 0.36, p < 0.01, Spearman’s rho = 0.28, p < 0.05, Fig. 4D).

## Discussion

To perceive a continuous and stable visual world despite the retinal image shifts that result from saccades, the visual system has to undergo some spatiotopic processing of the visual scene. In the present study, we confirmed that MST neurons maintained retinotopic RFs despite the saccades^25^. We further found that visual sensitivity after the saccades was modulated by the gaze angle to both the real visual stimuli and the visual memory traces remapped by the saccades. Neurons whose visual responses are modulated by the gaze angle (gain field neurons) were first reported in area 7a^16^,^17^ and then in several extrastriate visual areas in the dorsal visual stream of the monkey cortex^18^,^19^,^20^,^21^,^28^. Modeling studies hypothesized that this modulation functions as one of the steps involved in producing a spatiotopic map^22^,^24^. Thus, our finding that the remapped memory traces in the MST were modulated by gaze angle supports the concept of their role in constructing a continuous spatiotopic map of the visual scene despite the saccades.

The eye position gain modulation that we observed in the MST is conceptually similar to gaze-related modulation in the posterior parietal cortex, where the gain of visually evoked responses during fixation are modulated by ocular position^16^. We confirmed that the visual sensitivity of MST neurons was modulated by eye position when the monkey kept its eyes on a stationary target, as reported in a previous study^19^. Furthermore, we found that the amplitude of the post-saccadic responses of the MST neurons to a long duration visual stimulus (600 ms) were also modulated by eye position, while the location of the RFs moved with shifts in the eye position due to the saccades, resulting in the visual stimulus falling into the RFs. This result indicates that gaze-related modulation of the responses develops coincidentally with saccadic ocular shifts.

In our previous study, we recorded single unit activities in the MST while animals performed a saccade task. We found that after a saccade most MST neurons responded to a short-duration visual stimulus (170 ms) that had pre-existed inside of their post-saccadic RFs but turned off before the saccade (i.e., “memory remapping”)^25^. We confirmed the memory remapping of MST neurons, and found that the post-saccadic responses to the memory traces of the short-duration visual stimulus, which was visible only before the saccade, were also modulated by the post-saccadic eye position. This finding suggests that after the saccade the signal of the post-saccadic eye position affected the amplitude of the responses to the recalled information of the visual stimulus that had pre-existed in the post-saccadic RF but turned-off before the saccade. Thus, the eye position signal modulates the responses both to the actual stimulus and to the memory trace of the pre-existing-but-turned-off stimulus remapped by the saccade. Since the responses to the long-duration stimulus after the saccade comprise the remapped information and renewed information from the same visual stimulus^25^, the eye position signal should influence the neuronal responses after integration of the remapped and renewed information. Considering the role of the remapped visual memory traces in spatiotopic processing despite the saccades, the gain modulation of the memory response may closely correlate with both the modulation during fixation and that immediately after a saccade. Although the correlation between the GMIs during fixation and remapped responses was significant but not strong, it might be due to the fact that while the visual responses of MST neurons during fixation utilized the MT signal, which is already modulated by the eye position, the memory trace responses after saccades did not.

Sakata *et al*.^29^ recorded single unit activities in the anterior bank of the STS and found neurons activated by the fixation of a gaze on a stationary target; these neurons were classified as “visual fixation neurons” according to Mountcastle *et al*.^30^. They reported that the firing rates of most visual fixation neurons were modulated in relation to gaze angle. The area in which visual fixation neurons were recorded was subsequently called the MST following a paper written by Maunsell and van Essen^31^ (see Fig. 1 of Sakata, *et al*.^29^, Squatrito and Maioli^32^). The visual motion-sensitive MST neurons described in the present study might receive eye position information directly from the visual fixation neurons and/or inputs common to them. As suggested by Sakata *et al*.^29^, there are two possible sources that provide eye position signals to the MST: (1) “inflow” from proprioceptors; and (2) “outflow” of oculomotor signals (i.e., an efference copy or corollary discharge of the command to move the eye)^2^,^33^,^34^. The efference copy signal likely occurs simultaneously with or precedes the saccade. In contrast, the proprioceptive signal is known to occur after the change in eye position^35^. Since the gaze angle modulation began almost simultaneously with the arrival of the eye (50 ms at most after saccade offset), the origin of the eye position extra-retinal signal is most likely the efference copy of the command to extraocular muscles.

The main role of the gain field neurons in the posterior parietal cortex (e.g., LIP neurons) may be to represent the target position in spatiotopic coordinates and to guide quick, goal-directed motor actions such as saccades^18^. Xu *et al*.^35^ studied the time course of the eye position modulation of LIP visual responses to a briefly flashed target after a saccade, and observed unreliable modulation by the post-saccadic eye position for at least 150 ms after the saccade. They suggested that, in LIP, the gain field cannot be used to calculate the target position in space after the saccade; however, the gain field would provide feedback to recalibrate the efference copy signal after an eye movement or update a forward model to drive subsequent movements. In contrast, we observed that MST responses to both the real visual stimuli and the remapped memory traces were modulated by the post-saccadic eye position almost coincidently with the arrival of the eye. Thus, it is possible to calculate the stimulus position in space after the saccade. MST may thereby play a role in perceiving the continuous and stable visual world despite the saccades by employing the trans-saccadic memory (remapping) and the spatiotopic (gain field) mechanisms in close cooperation.

## Materials and Methods

### Animal preparation and physiological methods

Two male rhesus monkeys (Macaca mulatta) weighing 7 and 9 kg were used in this study. The Animal Care and Use Committee of Kyoto University approved all protocols, which complied with the guidelines established in the Public Health Service Guide for the Care and Use of Laboratory Animals (Approval number: MedKyo12033, 13037, 14045, 15535). The procedures used in the study were similar to those described elsewhere^25^.

**Figure 5:**
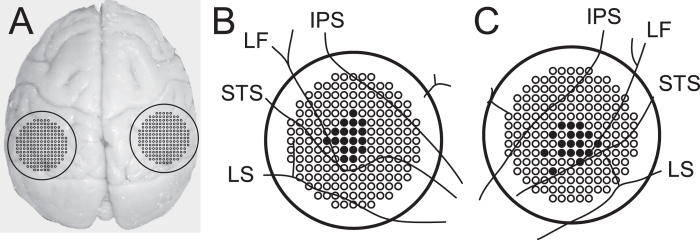
Sites of microelectrode penetration. (**A**) A photograph of the dorsal view of the cerebrum of one of the rhesus monkeys. The locations of the recording chambers and grid hole patterns are superimposed. The inside diameter of the chamber was 19 mm. (**B**,**C**) Sites of penetration guide tubes are plotted as filled black circles on the grid patterns and cerebral sulci ((**B**) left hemisphere; (**C**) right hemisphere). The center-to-center distance between the holes was 1 mm. IPS, intraparietal sulcus: LS, lunate sulcus; STS, superior temporal sulcus, LF, lateral fissure.

We studied neurons in MST within the dorsal part of the superior temporal sulcus (STS). The recording chambers were stereotaxically placed to allow for a dorsal approach to the posterior parietal cortex in the vertical orientation (stereotaxic coordinates: AP −2 to −4 mm, ML ± 16 to 18 mm (Fig. 5A). T1 magnetic resonance imaging (MRI) scans were used to confirm the location of the STS. Within the STS, MST neurons were identified based on their location relative to the STS and their RF characteristics^36^,^37^, see Supplemental Figure 1 in Inaba, *et al*.^38^ for histological reference.

### Recording technique and visual stimuli

The monkeys sat in a primate chair in a dark room and faced a cathode ray tube (CRT) monitor (FlexScan T766, Nanao, Japan), which was located 30 cm in front of the eyes, and performed saccade/fixation tasks. Visual stimuli were presented on the monitor (1280 × 1024 pixels (60° × 50°); refresh rate, 100 Hz). The period of exposure to the visual stimulus was monitored by a photo-diode (Sharp, SBC111) attached to the CRT monitor.

Initial mapping of the cortex in the dorsal part of the STS were made with hand-made glass-coated tungsten electrodes. Then, a guide-tube grid system (Crist Instruments^39^) was attached to the recording chamber (Fig. 5B,C) and single units were recorded with tungsten microelectrodes (Microprobe, FHC or Nano Biosensors) via transdural guide tubes inserted in the grid hole.

Having isolated a visual motion-sensitive neuron, we determined the neuron’s preferred direction by observing its responses to a moving random-dot pattern (with selection of a suitable field size for each neuron: 50° × 50° to 1.9° × 1.9°) in eight directions (horizontal, vertical and diagonal).

### Behavioral paradigms

In the fixation task, a stationary fixation target appeared on the CRT screen (Figs 1A and 2A,B). A moving sinusoidal grating pattern appeared when the monkey kept its eyes on the fixation target. The location of the fixation target was at the center or at 10° eccentricity on the horizontal or vertical axis (10° up and 10° down in the case of Fig. 2). The orientation of the grating pattern was orthogonal to the neuron’s preferred direction (either horizontal or vertical) (rightward arrows in Figs 1,2,3 and 4). The spatial frequency of the grating pattern was 0.6 cyc/°^26^. The grating stimulus always moved at 20°/s in the neuron’s preferred direction. Since the CRT screen was divided into 20 strips (either horizontal or vertical) to define the location of the RF, the grating pattern occupied 1 of the 40 strips, which was horizontal (60° wide and 2.5° tall, at V1–V20) (Fig. 1B) or vertical (3° wide and 50° tall, at H1–H20) (Fig. 1C). The moving grating and the fixation target were turned-off 600 ms after the onset of the moving grating. Until then, the monkey was required to keep its eyes within 1.5° of the target.

In the saccade task, a fixation target appeared at the center of the screen at the start of each trial (Figs 3A and 4A). After the monkey maintained fixation on the central target for 400 ms, a saccade target and a moving grating appeared at the same time as the central fixation target disappeared. The monkey had to make a saccade immediately after the central fixation target disappeared. The saccade target was located at 10° eccentricity on the horizontal or vertical axis (vertical in the case of Figs 3A and 4A). To reduce the effects of retinal motion induced by the saccade itself, the saccade axis was always orthogonal to the neuron’s preferred direction, which was parallel to the orientation of the grating pattern. The monkey was required to keep its eyes within 1.5° of the target until it disappeared. The duration of the exposure of the moving grating was either 600 ms (visible for the entire time period during and after the saccade, long-stimulus task, Fig. 3) or 170 ms (turned off before the saccade onset, short-stimulus task, Fig. 4). We aborted further recordings of the neuron when the center of its pre- or post-saccadic RF was outside the CRT monitor or when its border could not be defined, so that we could analyze neurons whose precise location of both the pre- and post-saccadic RF have been determined. Because the visual stimuli were presented on the CRT monitor, we could not make the room completely dark. Therefore the edges of the CRT in the peripheral field may have been visible even in the dark room. However, since the primary interest of the present study was to characterize the neuronal responses to the moving gratings, we did not attempt to further characterize the effect of the visibility of CRT-edges.

### Data collection and analysis

We exposed the animal to the stimulus conditions in a pseudo-randomized order. For all analyses, at least three repetitions of each stimulus condition (5.8 ± 1.7 trials: mean ± standard deviation) were required for each neuron. To evaluate the neuronal response, the firing rate of each neuron was averaged over a time period of 50–150 ms after the stimulus onset or 0–100 ms after the saccade offset. Spike-density functions were calculated by convolving the spike trains with a Gaussian of sigma 10ms^40^.

To characterize the neuron’s spatial tuning profile for each of the behavioral conditions, we quantified the neuronal responses to the moving stimulus at 20 locations. We fitted a Gaussian function involving four free parameters to the neuronal responses:


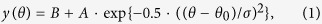


where B, A, θ_0_, and σ denote the baseline firing rate, maximal amplitude, optimal location, and tuning width, respectively^41^. The parameter values of the best-fit function were utilized to quantify the spatial tuning profiles.

To quantify the eye position effects on the neuronal responses, we calculated a gain modulation index (GMI) using the following expression:


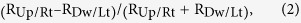


where R_Up_ (R_Rt_) denotes the mean firing rate of a neuron when the monkey fixated on a target located 10° up (10° right) from the center, R_Dw_ (R_Lt_) denotes the mean firing rate of a neuron when the monkey fixated on the target located 10° down (10° left) from the center. The index ranged from −1.0 to + 1.0; positive GMI values indicated that R_Up_ (R_Rt_) was greater than R_Dw_ (R_Lt_).

## Additional Information

**How to cite this article**: Inaba, N. and Kawano, K. Eye position effects on the remapped memory trace of visual motion in cortical area MST. *Sci. Rep*. **6**, 22013; doi: 10.1038/srep22013 (2016).
